# Neuroprotective Effects of Pre-Treament with l-Carnitine and Acetyl-l-Carnitine on Ischemic Injury *In Vivo* and *In Vitro*

**DOI:** 10.3390/ijms13022078

**Published:** 2012-02-15

**Authors:** Rui Zhang, Hong Zhang, Zhongxia Zhang, Tao Wang, Jingya Niu, Dongsheng Cui, Shunjiang Xu

**Affiliations:** 1 Central Laboratory, The First Hospital of Hebei Medical University, Shijiazhuang 050031, China; E-Mails: doudouzr511@yahoo.cn (R.Z.); smallrainn@gmail.com (Z.Z.); jyniu163@163.com (J.N.); dongshengcui@sina.com (D.C.); 2 Handan City Hospital for Infectious Diseases, Handan 056002, China; E-Mail: zhanghong_handan@sina.com; 3 Handan county Hospital, Handan 056002, China; E-Mail: wangtao9979@126.com

**Keywords:** ischemia, l-carnitine, Acetyl-l-carnitine, neuroprotection, oxygen-glucose deprivation

## Abstract

The therapeutic effect of stroke is hampered by the lack of neuroprotective drugs against ischemic insults beyond the acute phase. Carnitine plays important roles in mitochondrial metabolism and in modulating the ratio of coenzyme A (CoA)/acyl-CoA. Here, we investigate the neuroprotective effects of l-carnitine (LC) and Acetyl-l-carnitine (ALC) pre-treatment on ischemic insults under the same experimental conditions. We used a transient middle cerebral artery occlusion (MCAO) model to evaluate the protective roles of LC and ALC in acute focal cerebral ischemia *in vivo* and to understand the possible mechanisms using model of PC12 cell cultures *in vitro*. Results showed that ALC, but not LC, decreased infarction size in SD rats after MCAO *in vivo*. However, both LC and ALC pretreatment reduced oxygen-glucose deprivation (OGD)-induced cell injury and decreased OGD-induced cell apoptosis and death *in vitro;* at the same time, both of them increased the activities of super oxide dismutase (SOD) and ATPase, and decreased the concentration of malondialdehyde (MDA) *in vitro*. Thus, our findings suggested that LC and ALC pre-treatment are highly effective in the prevention of neuronal cell against ischemic injury *in vitro*, however, only ALC has the protective effect on neuronal cell injury after ischemia *in vivo*.

## 1. Introduction

**Figure 1:**
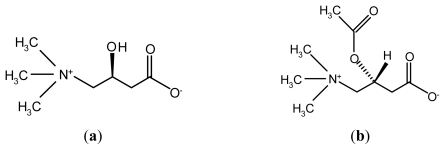
Chemical structure of l-carnitine (LC) (**a**) and Acetyl-l-carnitine (ALC) (**b**).

Ischemic stroke is considered as one of the leading causes of death and disability all over the globe [1]. A considerable proportion of strokes result from thromboembolic occlusion of major intracerebral vessels including the middle cerebral artery (MCA). Following focal cerebral ischemia, neuronal cell death is caused by both the core necrosis and the penumbra apoptosis within minutes to days [2–4]. The neuronal death after ischemia is closely linked to the essential role of mitochondrial metabolism. Cerebral ischemia leads to mitochondrial dysfunction due to lack of oxygen, leaving the glycolytic metabolism as a main pathway for energy production. Inhibition of mitochondrial respiratory chain triggers generation of lactate and hydrogen ions and dramatically reduces ATP generation leading to a dysregulation of cellular ion metabolism with subsequent intracellular calcium accumulation [5]. Although many efforts were made to develop novel drugs in order to rescue neurons from delayed neuronal death (DND) in the penumbral region, few drugs have shown efficacy currently in stroke patients [6]. Therefore we sought to elucidate the therapeutic benefits of prophylactic drugs to help reduce the severity of the stroke.

l-carnitine (4-*N*-trimethylamino-3-hydroxybutyric acid, LC) (Figure 1a) is an essential co-factor in the metabolism of lipids and consequently in the production of cellular energy [7]. This molecule plays important physiological roles in the β-oxidation of fatty acids by facilitating the transport of long-chain fatty acids across the mitochondrial inner membrane as acylcarnitine esters and the modulation of intracellular co-enzyme A homeostasis [8]. Acetyl-l-carnitine (ALC), an ester of LC (Figure 1b), serves as a source of acetylcholine and l-glutamate, and also contributes to energy-producing reactions. Several studies have suggested that LC and ALC may play a neuroprotective role in hypoxic-ischemic brain injury [9,10]. The early report demonstrated that LC significantly decreased infarct size compared with saline [11], but this result could not be replicated in the subsequent experiments. Recent studies have shown that LC pre-treatment ameliorated brain injury after hypoxia-ischemia in newborn rats through reducing apoptotic cell death and inhibiting the production of platelet-activating factor (PAF) [12]. Others studies have also shown ALC may be of benefit in reducing the infarct size and striatal glutamate outflow following focal cerebral ischemia in rats [13]. It is postulated from references that LC and ALC have different effects on different brain regions as well as different treatment methods of them have different effect on neuronal apoptosis. As ALC is an ester of LC, the question is which one is better to reduce the brain injury following focal cerebral ischemia? We have not seen the comparative study for LC and ALC under the same experimental conditions to clarify its neuroprotective effects against ischemic injury.

This study was therefore designed to determine the effects of LC and ALC administration on brain infarct size under the same experimental conditions *in vivo* and to understand the possible mechanism using *in vitro* model of PC12 cell cultures.

## 2. Results

### 2.1. ALC, But Not LC, Decreases Infarction Size in SD Rats after MCAO

**Figure 2:**
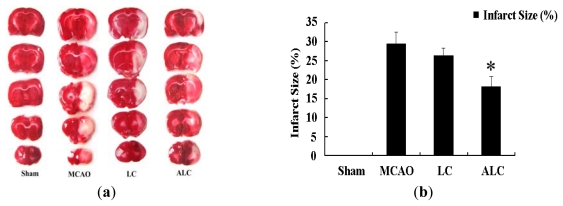
Effects of LC and ALC on infarct size following middle cerebral artery occlusion (MCAO) in rats. (**a**) 2,3,5-triphenyltetrazolium chloride (TTC) staining of cerebral infarct size in brain sections from sham groups, MCAO groups, LC (100 mg/kg) and ALC (100 mg/kg) pretreated groups at 24 h before MCAO; (**b**) Quantification of infarct size by TTC staining 6 h after MCAO. Values are mean ± SEM. * *p* < 0.05 compared to the MCAO groups, *n* = 6 in each group.

To illuminate the protective effects of LC and ALC in acute focal cerebral ischemia, we performed MCAO in male SD rats. MCAO model group rats received intraperitoneal injection of PBS only, as well as LC and ALC group rats received intraperitoneal injection of LC or ALC (100 mg/kg) 24 h prior to MCAO, respectively. Animals were sacrificed 6 h after MCAO or sham surgery for 2,3,5-triphenyltetrazolium chloride (TTC) staining. A typical TTC staining is shown in Figure 2a. No infarction was found in any rats with sham surgery group. ALC (*n* = 6) pre-treatment obviously decreased the infarction size of cortical infarction compared to PBS pretreatment (*n* = 5) (*p* < 0.05). However, LC pre-treatment had no significant effect on infarct size compared to control group (*p* > 0.05, Figure 2a,b).

### 2.2. LC and ALC Pretreatment Reduces Oxygen-Glucose Deprivation-Induced Cell Injury

**Figure 3:**
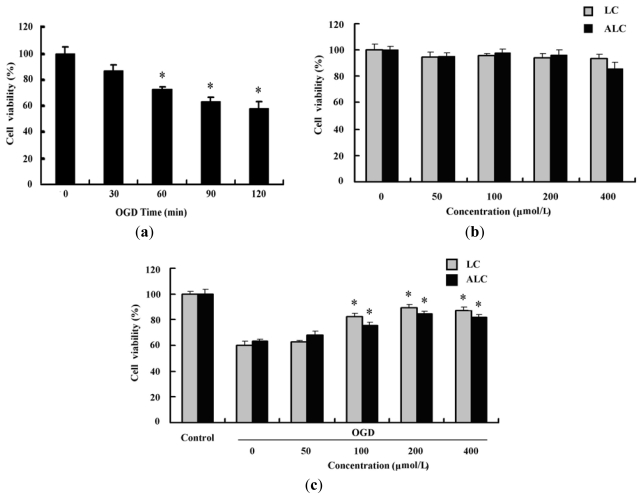
Effects of LC and ALC pretreatment on the viability of PC12 cells exposed to oxygen-glucose deprivation (OGD). (**a**) OGD decreased the viability of PC12 cells time-dependently. PC12 cells were treated with OGD for 30, 60, 90, and 120 min, and the cell viability was determined by MTT assay (*n* = 6); (**b**) Effect of LC and ALC on the viability of PC12 cells treated without OGD. Cells were pretreated with 50, 100, 200 or 400 μmol/L LC and ALC for 24 h, and the cell viability was determined by MTT assay (*n* = 6); (**c**) Dose-dependent increase of the cell viability by LC and ALC in PC12 cells treated with OGD. Cells were pretreated with 50, 100, 200 and 400 μmol/L LC or ALC for 24 h, followed by OGD exposure for 120 min, and the cell viability was determined by MTT assay (*n* = 6). Values are mean ± SEM. * *p* < 0.05 compared to the OGD groups.

To elucidate the different protective role of LC and ALC on the infarction size in rats after MCAO, oxygen-glucose deprivation (OGD)-induced cell injury, a popular neuronal cell injury model *in vitro* was used. We firstly detected the effect of OGD on PC12 cells. After the PC12 cells were incubated in hypoxic conditions in the absence of glucose for 30, 60, 90, and 120 min, the effects of such conditions on the cell viability were determined. The results showed that OGD caused progressive reduction in the cell viability time-dependently as determined by thiazolyl blue terazolium bromide (MTT) assay (*p* < 0.05, Figure 3a). After a 120 min exposure to OGD, the cell viability had dropped to ~60%. The effects of LC and ALC on the cell viability of non-OGD PC12 cells were also observed. The results showed that the cell viability of the non-OGD PC12 cells were not affected by pretreatment with LC and ALC (*p* > 0.05, Figure 3b). However, both LC and ALC pretreatment increased dose-dependently the viability of PC12 cells exposed to OGD (*p* < 0.05, Figure 3c).

### 2.3. Pretreatment with LC or ALC Decreases OGD-Induced Cell Apoptosis and Death

**Figure 4:**
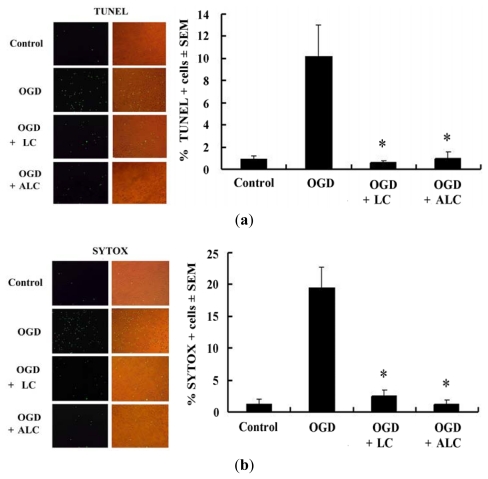

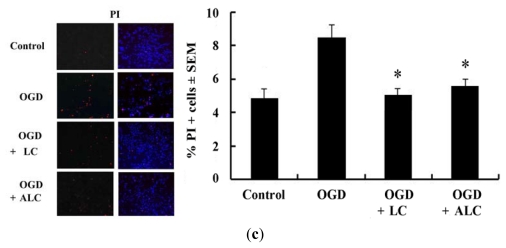
Effects of LC and ALC pretreatment on apoptosis and necrosis of PC12 cells treated with OGD. PC12 cells were pretreated without or with LC (200 μmol/L)) and ALC (200 μmol/L) for 24 h before OGD exposure for 120 min. PC12 cells treated without OGD used as an normal control. (**a**) Cell apoptosis were examined by TUNEL fluorescence staining; (**b**) Cell necrosis were examined by SYTOX fluorescence staining; (**c**) Cell necrosis were examined by PI fluorescence staining. * *p* < 0.05 compared to the OGD groups. Original magnifications: 200×.

To further illuminate the possible mechanisms responsible for the increase in OGD-induced PC12 cell viability by LC and ALC pre-treatment, the cell apoptosis and death rate were detected by TUNEL, SYTOX, and PI staining, respectively. The positive stained cells were calculated by counting 5 randomly selected fields, and results were expressed as % positive cells/total cells ± SEM. As shown in Figure 4, the percentage of positive TUNEL cells induced by OGD was significantly reduced by LC and ALC pre-treatment (*p* < 0.05), suggesting that LC and ALC inhibit OGD-induced PC12 cell apoptosis (Figure 4a). The percentage of positive SYTOX and PI stained cells were calculated as above. The results showed that the percentage of positive SYTOX and PI cells induced by OGD was significantly reduced by LC and ALC pre-treatment (*p* < 0.05), suggesting that LC and ALC inhibit OGD-induced PC12 cell death (Figure 4b, c). Taken together, LC or ALC pre-treatment increased the cell viability by decreasing OGD-induced cell apoptosis and death.

### 2.4. Effects of LC or ALC on the SOD, MDA, and ATPase in OGD-Induced PC12 Cells

**Figure 5:**
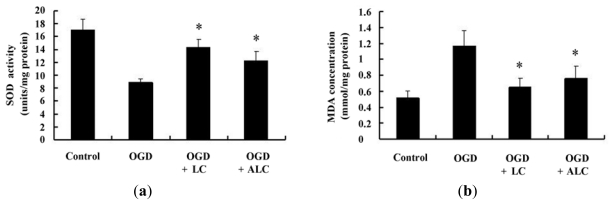

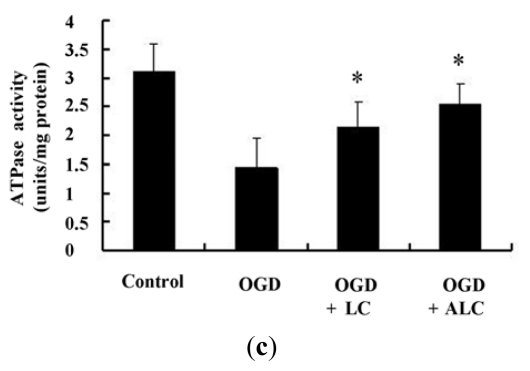
Effects of LC and ALC pretreatment on the activity of SOD and ATPase, and the level of MDA in PC12 cells exposed to OGD. PC12 cells were pretreated without or with LC (200 μmol/L) and ALC (200 μmol/L) for 24 h before OGD exposure for 120 min. PC12 cells treated without OGD used as an normal control. (**a**) Super oxide dismutase (SOD) activity in PC12 cells; (**b**) The levels of MDA in PC12 cells; (**c**) The ATPase activity in PC12 cells. Values given are the mean ± SEM (*n* = 6). * *p* < 0.05 compared with OGD groups.

Because super oxide dismutase (SOD) is considered as a primary antioxidant defense in cells, we observed the SOD activity in the PC12 cells to determine whether LC and ALC had regulatory effects on SOD activity after OGD. The results showed that LC or ALC pre-treatment increased the SOD activity after OGD (*p* < 0.05). ATPase, which involves in fluid, electrolyte, and nutrient transport [14], was also measured. As shown in Figure 5, the incubation of PC12 cells in OGD conditions could decreased obviously the ATPase activity (*p* < 0.05). When the cells were pretreated with LC or ALC for 24 h, the ATPase activity was significantly increased in OGD PC12 cells (*p* < 0.05). The levels of malondialdehyde (MDA), which is a byproduct of a free radical attack on lipids, increased significantly after PC12 cells exposure to OGD compared to normal control. LC or ALC pre-treatment for 24 h could decrease MDA levels significantly in OGD PC12 cells (*p* < 0.05).

## 3. Discussion

LC and ALC cross the blood-brain barrier and are widely distributed in the central nervous system. They have wide roles in brain metabolism [7]. The previous study showed that carnitine has a protective effect on hypoxia-ischemia injury [9]. Moreover, carnitine play essential roles in inhibiting glutamate-mediated toxicity *in vivo* via decreasing the affinity of glutamate to its receptor and increasing glutamate transport [15,16]. Due to different isomers or modification of the drugs play different roles in medical treatment [17], the effects and underlying mechanisms of LC and its acetyl ester ALC on the infarction size in rats after MCAO, were tested in the same experimental conditions. Clinically, stroke patients need to receive most effective therapy within 6 h. Consequently, in the present study, we focused on the effects of LC and ALC pretreatment on the infarct size at 6 h after MCAO. We found that pretreatment with ALC, but not LC, decreased the infarction size significantly in rats after MCAO, suggesting that ALC was better to reduce the brain injury following focal cerebral ischemia. Plasma concentration of free LC is in dynamic balance with ALC and both of them are actively transported into cells via the organic cation/carnitine transporter (OCTN) system [18]. It is reported that OCTN2 is functionally expressed in rat astrocytes, and is responsible for LC and ALC uptake in these cells. The subsequent kinetic analysis showed the Michaelis-Menten constant of LC was higher than that of ALC, suggesting that the affinity of LC to its transporter is lower than that of ALC [19]. So it is postulated that the different affinity of LC or ALC might play an important role in exerting its neuroprotective effects on ischemic injury. A most recent report suggested that ALC elevate the glucose uptake and the glucose transporter protein expression level, and the acetyl moiety of ALC is metabolized for energy in both astrocytes and GABAergic neurons, which may also contribute to its effect of reducing neurotoxicity and neuronal degeneration [20,21]. To elucidate the different mechanisms of LC and ALC on the infarction size in rats after MCAO, an *in vitro* ischemic model (oxygen glucose deprivation, OGD) in PC12 cells were used as reported previously [22]. It is well known that PC12 cells are the most widely used cellular model for studying neuronal diseases including Alzheimer, Parkinson, stroke, and ischemia. In the present study, *in vitro* ischemia (OGD) was delivered by incubating PC12 cells with a medium in which glucose was omitted and oxygen was replaced with N2. The viability of PC12 cells decreased progressively when exposed to OGD for different times, which indicated that OGD could induce ischemic injury under our experimental conditions. It is reported that ALC played a role in neuroprotection during hypoxia, which is attributed to mitochondrial biosynthesis regulated by ERK1/2-Nrf2 signaling [23]. We found that LC or ALC itself did not affect the viability of PC12 cells under normal cultural conditions, but pre-incubation with LC or ALC increased the PC12 cell viability when exposed to OGD. Our data suggest that LC and ALC both are capable of protecting the PC12 cells from OGD-induced damage *in vitro*.

Lately, it is reported that necrotic and apoptotic cell death of many neurons in the ischemic areas was found after a stroke event [24]. We hypothesized that LC and ALC would increase the cell viability by decreasing OGD-induced cell apoptosis and death. In agreement with this hypothesis, our studies *in vitro* showed that LC and ALC reduced the cell apoptosis and death rate by TUNEL, SYTOX, and PI staining, respectively. It is indicated that the reduction of cell apoptosis and death rate by LC or ALC might contribute to its neuroprotective role following *in vitro* ischemia.

Oxidative stress has been involved in the pathogenesis of several neurological diseases including acute stroke. Dysfunctional mitochondria exposed to oxygen in the penumbral area of the infarct generate higher amounts of reactive oxygen species (ROS) than can be scavenged by the antioxidant systems, leading to oxidative stress [25]. Ischemic damage is induced by the accelerated formation of reactive oxygen species including superoxide and nitric oxide (NO) radicals. These ROS can damage cellular components such as proteins, lipids, and DNA, resulting cell apoptosis and death. Recent reports demonstrated that LC supplementation has a protective role on exhaustive exercise-induced oxidative stress [26]. Previous studies have demonstrated that SOD has a significant role in neuroprotection in nature [27], and ALC may lead to SOD stabilization [28].

Moreover, as is well known, carnitine prevents free radical formation via inhibiting the activity of enzymes involving their generation and via inducting antioxidant mechanisms [29]. Our study showed the activity of SOD was significantly decreased in OGD PC12 cells as compared to non-OGD cells, and both LC and ALC significantly prevented the decrease of the activity of SOD. The same changes did occur in the activity of ATPase. In addition, we also estimated the content of MDA, which is a byproduct of a free radical attack on lipids. These results suggested that LC and ALC have the neuroprotective effect by improving the state of oxidative stress during ischemic injury.

Although the exact mechanisms responsible for the inhibition of ischemic injury by ALC *in vivo* are still unclear at this time, at least, our results provide the evidences that LC and ALC pre-treatment are highly effective in the prevention against neuronal cell injury *in vitro* in the same test condition, which is consistent with a previous report [30]. Based on our results, we speculated that the different pharmacokinetics, mechanism and time of action may result in the different effects of LC and ALC on ischemic injury *in vivo*. Thus, further studies will be required to elucidate the exact mechanisms.

## 4. Experimental Section

### 4.1. Experimental Animals

All experimental procedures were approved by the Animal Care and Use Committee of Hebei medical University. Male Sprague-Dawley rats weighing 300 ± 30 g were kept under constant environmental conditions (temperature, 22 ± 2 °C; humidity, 55 ± 5%; 12-/12-h light/dark cycle) in the Animal Research Center of Hebei medical University. Before and after all experimental procedures, all animals had free access to food and water. Before the experimental procedures, all animals were clinically normal and without infection or inflammation, and had no neurological deficits. Rats were divided into four groups (sham, MCAO, LC and ALC group). Anesthesia was induced by 4.5% halothane in N_2_O:O_2_ (70%:30%) and was maintained by inhalation of 1.5% halothane by mask.

### 4.2. Transient Middle Cerebral Artery Occlusion (MCAO)

An intraluminal filament model was used to induce transient MCAO. Briefly, through a midline neck incision, the right common, external, and internal carotid arteries were dissected and exposed. The common and external carotid arteries were ligated by sutures. A silicone-coated nylon filament (diameter: 0.37 mm, Doccal Corporation, Redlands, CA, USA) was then inserted into the external carotid artery and gently advanced into the internal carotid artery, until mild resistance was felt, indicating that occlusion of the origin of the middle cerebral artery in the Willis circle was achieved. The wound was sutured, and the rats were returned to their cages. To understand the protective effects of LC and ALC (Sigma, St. Louis, MS, USA) on MCAO model, intraperitoneal injection of LC or ALC (100 mg/kg) 24 h before MCAO. MCAO group rats received intraperitoneal injection of PBS only.

### 4.3. Measurement of the Infarct Size

All animals were decapitated 6 h after MCAO. Their brains were quickly removed, placed in cold saline solution for 10 min, and then cut into 2-mm-thick coronal slices using a rodent brain matrix. Five selected sections were stained in a 2% solution of TTC (Sigma, St. Louis, MS, USA) at 37°C for 30 min. Areas ipsilateral to the occlusion, which could not be stained, were recorded as infarcted. After the sections were fixed with 10% formaldehyde, infarct size was measured by using an imaging system (AIS, Imaging Research Inc.), The corrected infarct size was calculated by using an indirect method in order to compensate for the effect of brain edema [31].

### 4.4. Cell Culture and Treatments

Rat pheochromocytoma (PC12) cells (American Type Culture Collection, Rockville, MD, USA) were cultured in Dulbecco’s modified Eagle’s medium (DMEM) (Invitrogen, Carlsbad, CA, USA) supplemented with 10% horse serum, 5% fetal bovine serum (FBS), 100 kU/L of penicillin, and 100 mg/L of streptomycin (Sigma, St. Louis, MS, USA) at 37 °C with a 5% CO_2_ atmosphere in a humidified incubator. All treatments were performed on cells at 60% to 80% confluence.

### 4.5. Oxygen-Glucose Deprivation (OGD)

In order to mimic ischemic-like conditions *in vitro*, PC12 cells were incubated with glucose-free buffer contained 154 mmol/L NaCl, 5.6 mmol/L KCl, 3.6 mmol/L NaHCO_3_, 2.3 mmol/L CaCl_2_ and 5.0 mmol/L HEPES at 37 °C for 15 min in an oxygen-free N_2_/O_2_ (95%/5%) atmosphere, and then the chamber was sealed and the cells were culture in the hypoxic conditions for 30, 60, 90, and 120 min. After incubation for 120 min, cells were removed from the anaerobic chamber to normal culture conditions. The cells were divided into control, OGD, OGD plus LC pretreatment and OGD plus ALC pretreatment groups. Glucose (1.0 g/L) was added to make glucose containing buffer. The control group were incubated in glucose containing buffer, and maintained under normoxic conditions. Other groups which pretreated with PBS (OGD group), LC (200 μmol/L) or ALC (200 μmol/L) for 24 h, respectively, were exposed to OGD for 3 h.

### 4.6. Cell Viability Assay

Cell viability was measured by MTT assay. PC12 cells were seeded at 1 × 10^4^ cells/well in 96-well plates and allowed to grow in the DMEM medium for 24 h. After LC/ALC and OGD treatment, cells were incubated with 5 mg/mL MTT (Sigma, St. Louis, MS, USA) for 4 h, and subsequently solubilized in DMSO. The absorbency at 570 nm was then measured using an enzyme-linked immunosorbent assay (ELISA) reader. Experiments were repeated at least three times, and the data were expressed as the means ± SEM.

### 4.7. Measurement of Cell Death and Apoptosis

The TUNEL method was used to identify the DNA fragmentation of apoptotic cells. The procedure was performed following the manufacturer’s instruction (Roche, Basel, Switzerland). Briefly, PC12 cells were fixed with 4% paraformaldehyde for 30 min and washed with PBS 3 times. Then the cells were incubated with 3% H_2_O_2_ in 70% methanol for 10 min and washed with PBS twice. After that, the cells were incubated with 0.1% Triton X-100 in 0.1% sodium citrate for 2 min on ice. The slides were washed with PBS twice and the areas around specimens were dried. Subsequently, TUNEL reaction mixture (50 μL) was added to each sample. And then, slides were incubated in a humidified atmosphere for 60 min at 37 °C without light. After washing with PBS 3 times, stained sections were analyzed with fluorescence microscopy (Olympus, Tokyo, Japan). SYTOX green (Invitrogen, Springfield, OR, USA) and propidium iodide (PI, Sigma-Aldrich, St. Louis, MO, USA) were used to stain the cells with disrupted membranes (dead cells). Briefly, for SYTOX green staining, SYTOX green (25 nmol/L) was added to the PC12 cell cultures. After 10 min at room temperature, specimens were observed with a fluorescence microscopy. For PI staining, PC12 cells were incubated in the PI staining solution (5 μg/mL in 10 mmol/L PBS) for 10 min at room temperature in the dark. Then, the specimens were washed with PBS and mounted on slides. All the specimens were observed with a microscope. Thus, this assay includes apoptotic, dead, and viable cells.

### 4.8. Measurement of SOD, ATPase Activities and MDA Content

Neuroprotective effect of LC and ALC was confirmed in terms of excitotoxicity (ATPase activity) and oxidative stress (malondialdehyde, MDA; super oxide dismutase, SOD) in the PC12 cells. Briefly, total protein was isolated and the protein content was determined with the Bio-Rad Bradford protein assay. SOD, ATPase activities and content of MDA assays were performed using the commercial assay kits (Jiancheng Institute of Biotechnology, Nanjing, China), following the manufacturer’s protocols.

### 4.9. Statistical Analysis

All data are expressed as mean ± SEM. One-way ANOVA and Duncan’s multiple comparison test of the means were done using the software SPSS 16.0 [32] to compare the data obtained. A *p*-value < 0.05 was considered statistically significant.

## 5. Conclusions

In this work, we studied the effects of carnitines, LC and ALC, in protecting hypoxia-ischemia injury. The principal findings of this study can be summarized as follows: (1) ALC pre-treatment decreased the infarct size in the model of MCAO, however, LC pre-treatment had no effect on the infarct size in the model of MCAO; (2) both LC and ALC pre-treatment reduced OGD-induced PC12 cell injury *in vitro*; (3) both LC and ALC pre-treatment decreased OGD-induced PC12 cell apoptosis and death *in vitro*. These findings suggested that LC and ALC pre-treatment are highly effective in the prevention of neuronal cell against ischemic injury *in vitro;* however, only ALC pre-treatment has a protective effect on neuronal cell injury after ischemia *in vivo.*
